# Supplementary material for: Patient priorities in relation to surgery for gastric cancer: qualitative interviews with gastric cancer surgery patients to inform the development of a core outcome set
Source: BMJ Open. 2020 Feb 12;10(2):e034782. doi: 10.1136/bmjopen-2019-034782 (PMC7044961; doi:10.1136/bmjopen-2019-034782)
Supplement: Supplementary data [file bmjopen-2019-034782supp001.pdf]

Appendix 1. Development of themes

Theme: Surviving and Controlling Cancer

Issues identified on initial coding	Outcome	Outcome theme
Being cured of cancer<br>Cancer returning to other parts of the body<br>Cancer returning in the abdomen<br>Possibility of cancer returning	Recurrence of Cancer	Surviving and Controlling Cancer
Being able to live (a little/a lot) longer<br>Being alive/surviving for a 'long time'<br>Chances of (not) dying from cancer<br>Chances of survival	Survival	

Theme: Adverse events following surgery

Issues identified on initial coding	Outcome	Outcome theme
Complications related to anaesthesia	Anaesthetic complications	Adverse events following of Surgery
Anastomotic leak<br>Anastomotic stricture	Anastomotic complications	
Internal bleeding requiring further intervention	Bleeding	
Concern about cardiac complications in context of previous myocardial infarction<br>Racing heart beat	Cardiac complications	
Stroke following surgery	Cerebro-vascular complications	
Bowel perforation<br>Gastro-intestinal symptoms e.g. constipation<br>Obstruction of bowel	Intestinal complications	
Epidural related complications<br>Hallucinations<br>Overdose of medications such as morphine<br>Side effects of sedatives	Medication related complications	
Drains and tubes to manage complications<br>Endoscopic treatment of anastomotic stricture<br>Requiring further surgery to manage complications	Need for re-intervention	

Surgery for incisional hernia		
'Surviving' surgery<br>Dying from a complication of surgery<br>Dying on the operating table	Peri-operative death	
Hospital acquired pneumonia<br>Pleural effusion<br>Pneumothorax	Respiratory complications	
Re-admission due to complications such as infections<br>Re-admission due to pain	Re-admission to hospital	
Wound dehiscence<br>Wound infection<br>Wound leak<br>Wound numbness	Wound complications	
Catheter-related problems	Urinary complications	

Theme: Long-term impact of surgery

**Issues identified on initial coding**	**Outcome**	**Outcome theme**
Being able to enjoy a good quality of life<br>Uncertainty as to what life will be like following surgery	Overall 'quality of life'	Long-term impact of surgery
Changes in mood<br>Clinical depression<br>Feeling 'abnormal' and 'different' to others<br>Feelings of insecurity<br>Feelings of isolation<br>Issues related to body image<br>Low mood	Psychological impact	
Being able to enjoy eating again<br>Being able to exercise again<br>Being able to interact and socialise with others<br>Being able to live 'as they did before'<br>Being able to rely on oneself to undertake tasks<br>Being able to undertake household activities	Returning to 'normality'	

such as shopping and gardening<br>Returning to employment		

Theme: Technical aspects of surgery

**Issues identified on initial coding**	**Outcome**	**Outcome theme**
'Cutting' the cancer out<br>Ensuring no cancer is left behind<br>Getting 'rid' of the cancer<br>Inability to resect cancer at surgery<br>Removing all lymph nodes<br>Removing spleen if necessary	Complete resection of cancer	Technical aspects of surgery
Ability to perform laparoscopic 'keyhole' surgery<br>Large scars	Size of incisions	
Duration of surgery	Duration of surgery	

Theme: Long-term problems following surgery

**Issues identified on initial coding**	**Outcome**	**Outcome theme**
Amounts able to eat and drink<br>Being able to eat 'properly'<br>Being able to eat at home<br>Change in diet and types of food patient can consume<br>Difficulties swallowing	Eating and Drinking	Long-term problems following surgery
Requirement for ongoing nutritional support<br>Vitamin B12 deficiency	Nutritional problems	
Feeling persistently tired<br>Feeling extremely weak/lethargic/tired<br>Having no energy or stamina<br>Loss of energy following simple tasks	Fatigue	
Abdominal bloating<br>Belching<br>Diarrhoea<br>Dumping syndrome<br>Excessive flatus<br>Nausea	Gastro-intestinal symptoms	

Reflux symptoms (acid or bile)<br>Vomiting		
Abdominal pain or cramps<br>Headaches and migraines<br>Long-term wound related pain<br>Muscle cramps<br>Pain on swallowing<br>Painful abdominal distension or bloating	Chronic Pain	
Inability to regain weight to desired level<br>Readjusting to new weight<br>Speed of weight loss<br>Weight loss in general	Weight problems	

Theme: Recovery Following surgery

**Issues identified on initial coding**	**Outcome**	**Outcome theme**
Post-operative plan for physiotherapy<br>Restricted mobility due to drains and tubes attached<br>Time to be able to undertake tasks such as standing up, walking or bathing	Ambulation	Recovery following surgery
Time before being allowed to eat and drink<br>Time before bowel function returned	Return of gastrointestinal function	
Concern about being too unwell for further chemotherapy	Ability to have more chemotherapy	
Length of time in hospital<br>Length of time in intensive care	Duration of hospital stay	
Length of time in pain<br>Patterns of pain<br>Requirement for analgesia<br>Severity of pain	Post-operative pain	
