# Supplementary material for: Patient priorities in relation to surgery for gastric cancer: qualitative interviews with gastric cancer surgery patients to inform the development of a core outcome set
Source: BMJ Open. 2020 Feb 12;10(2):e034782. doi: 10.1136/bmjopen-2019-034782 (PMC7044961; doi:10.1136/bmjopen-2019-034782)
Supplement: Supplementary data [file bmjopen-2019-034782supp002.pdf]

Appendix 2. Summary of outcomes and outcome themes identified from interviews.

Outcome Theme	Outcome	How many interviews outcome was referenced in	Total number of references in all interviews
**Surviving and controlling cancer** • Referenced in 20 interviews • Referenced 90 times in all interviews.	Curing Cancer	4	6
	Recurrence of Cancer	18	28
	Survival	20	56
**Technical aspects of surgery** • Referenced in 18 interviews • Referenced 52 times in all interviews.	Complete Excision of Cancer	18	52
	Excision of Lymph Nodes	5	5
	Need for splenectomy	1	1
	Operative time	1	2
	Wound Size	7	11
**Adverse events** • Referenced in 20 interviews • Referenced 97 times in all interviews.	Ability to have adjuvant chemotherapy	1	1
	Anaesthetic Complications	1	1
	Anastomotic Leak	6	9
	Anastomotic Stricture	1	1
	B12 Deficiency	5	5
	Bleeding	1	2
	Cardiac Complications	2	2
	Catheter related complications	1	1
	Cerebro-vascular complications	1	1
	Gastrointestinal problems	1	1
	Hernia	1	1
	Intestinal complications	4	10
	Medication-related complications	10	12
	Need for reintervention	8	13

	Peri-operative death	12	20
	Re-Admission to Hospital	3	4
	Respiratory complications	3	3
	Wound Complications	8	10
**Recovery from surgery**<br>• Referenced in 18 interviews<br>• Referenced 57 times in all interviews.	In Hospital Recovery	11	23
	Length of Stay Following Surgery	11	18
	Peripheral Oedema	1	1
**Long-terms problems following surgery**<br>• Referenced in 20 interviews<br>• Referenced 175 times in all interviews.	Eating & Drinking	20	75
	Fatigue	16	38
	Gastrointestinal symptoms	11	27
	Pain	10	14
	Weight Loss	12	21
**Long-term impacts of surgery**<br>• Referenced in 20 interviews<br>• Referenced 133 times in all interviews.	Necessity of long-term feeding	1	1
	Overall QoL	8	10
	Psychological impact	11	40
	Returning to normality	20	82
